# Supplementary material for: Variability of Genetic Characters Associated with Probiotic Functions in Lacticaseibacillus Species
Source: Microorganisms. 2022 May 13;10(5):1023. doi: 10.3390/microorganisms10051023 (PMC9145642; doi:10.3390/microorganisms10051023)
Supplement: Supplementary file 1 [file microorganisms-10-01023-s001.zip › microorganisms-1687073-supplementary.pdf]

Table S1. Genes encoding functions required for survival/persistence in GIT, adhesion and exopolysaccharides production in the genome of *L. rhamnosus* GG (GenBank Acc. n. NC_013198.1/FM179322.1) and their presence/absence, identity and coverage percentages in other fully annotated *Lacticaseibacillus* genomes.

Gene/Gene Cluster	Function	% Genomes with Homologous Genes	% Identity Range	% Query Coverage Range
LGG_00031	p40 peptidoglycan hydrolase, Surface antigen	*L. casei*: 100	79.17–82.71	100
		L. paracasei: 100	77.46–79.33	100
		L. rhamnosus: 100	98.88–100	100
		L. zeae: 100	82.31–82.94	100
LGG_00051	GalC Possible cell wall associated protein with FIVAR domain, putative binding domain with hyaluronate or fibronectin as possible substrates	*L. casei*: 60	74.08–73.67	98
		L. paracasei: 0		
		L. rhamnosus: 85	100–95.04	100
		L. zeae: 100	73.71–73.63	96–98
LGG_00107	RmlC, dTDP-4-dehydrorhamnose 3,5-epimerase	*L. casei*: 0		
		L. paracasei: 0		
		L. rhamnosus: 92	98.82–100	100
		L. zeae: 0		
LGG_00172	TauB, taurine uptake system *tau*ABC	*L. casei*: 0		
		L. paracasei: 50	73.16–73.33	77
		L. rhamnosus: 54	99.18–100	100
		L. zeae: 0		
LGG_00173	TauA, taurine uptake system *tau*ABC	*L. casei*: 0		
		L. paracasei: 50	70.62–70.76	51
		L. rhamnosus: 54	98.18–100	100
		L. zeae: 0		
LGG_00174	TauC, taurine uptake system *tau*ABC permease	*L. casei*: 0		
		L. paracasei: 50	72.33–75.25	62–78
		L. rhamnosus: 54	99.62–100	100
		L. zeae: 0		
LGG_00183	YqcC C-terminal WxL domain	*L. casei*: 100	71.18–70.17	74–85
		L. paracasei: 100	70.20–70.78	62
		L. rhamnosus: 100	97.27–100	100

		L. zeae: 100	69.14–69.19	83	
LGG_00278	O-antigen ligase family protein	*L. casei*: 0			
		L. paracasei: 30			
		L. rhamnosus: 45	100	100	
		L. zeae: 0			
LGG_00279	WelA, dTDP-rhamnosyl transferase RfbF	*L. casei*: 0			
		L. paracasei: 30	67.71–71.26	30–70	
		L. rhamnosus: 32	100	100	
		L. zeae: 0			
LGG_00280	WelB, alpha-L-Rha alpha-1,3-L-rhamnosyltransferase	*L. casei: 0*			
		L. paracasei: 29	66.29–73.26	89–90	23–70
		L. rhamnosus: 32	100	100	100
		L. zeae: 0			
LGG_00281	WelC, alpha-L-Rha alpha-1,3-L-rhamnosyltransferase	*L. casei*: 0			
		L. paracasei: 95.8	69.03–69.41	76	
		L. rhamnosus: 32	100	100	
		L. zeae: 0			
LGG_00282	Eps1, Polysaccharide Transporter, PST family protein	*L. casei*: 40	62.75–69.73	37–82	
		L. paracasei: 53	62.26–74.12	30–98	
		L. rhamnosus: 32	9.93–100	100	
		L. zeae: 0			
LGG_00283	Eps2, CpsH	*L. casei*: 0			
		L. paracasei: 26	70.84–71.86	94–98	
		L. rhamnosus: 32	99.90–100	100	
		L. zeae: 0			
LGG_00295	Glycosyltransferase, group 2	*L. casei*: 100	78.48–78.87	99	
		L. paracasei: 100	73.88–74.71	99	
		L. rhamnosus: 100	97.57–100	100	
		L. zeae: 100	77.84	99	
LGG_00324	p75 cell wall peptidase, surface antigen	*L. casei*: 100	77.4–78.89	67–74	
		L. paracasei: 100	78.63–79.30	66–86	
		L. rhamnosus: 100	96.26–100	100	

		L. zeae: 100	76.35–76.5	67
LGG_00348	YohJ, lypopolysaccharide biosynthesis protein	*L. casei*: 0<br>*L. paracasei*: 32.7<br>*L. rhamnosus*: 100<br>*L. zeae*: 0	 68.37–70.58<br>97.71–100	 91–98<br>100
LGG_00349	YohH, Polyglycerol-phosphate alpha-glucosyltransferase, Lipopolysaccharide biosynthesis protein, glycosyltransferase activity	*L. casei*: 100<br>*L. paracasei*: 32.7<br>*L. rhamnosus*: 100<br>*L. zeae*: 100	68.07–70.92<br>74.35–74.60<br>97.15–100<br>66.90–69.48	30–52<br>99<br>100<br>48–58
LGG_00422	Gram-positive pilin subunit D1, SpaA, LPXTG cell wall anchor motif	*L. casei*: 0<br>*L. paracasei*: 100<br>*L. rhamnosus*: 27<br>*L. zeae*: 0	 98.61–99.80<br>97.21–100	 100<br>100
LGG_00434	NagZ, β-N-acetylhexosaminidase, LPXTG cell wall anchor domain	*L. casei*: 100<br>*L. paracasei*: 0<br>*L. rhamnosus*: 33<br>*L. zeae*: 100	68.97–69.33<br>88.87–100<br>100<br>68.78–68.81	66<br>99–100<br>100<br>66
LGG_00441	SrtC1 sortase	*L. casei*: 0<br>*L. paracasei*: 100<br>*L. rhamnosus*: 27<br>*L. zeae*: 100	 98.11–99.35<br>97.96–100	 97–100<br>99–100
LGG_00442	SpaA, pilus specific protein, major backbone protein,<br>LPXTG cell wall anchor domain-containing protein	*L. casei*: 0<br>*L. paracasei*: 100<br>*L. rhamnosus*: 27<br>*L. zeae*: 0	 98.86–9.20<br>97.21–100	 100<br>100
LGG_00443	SpaB, Pilus specific protein, minor backbone protein,<br>Gram-positive pilin subunit D1	*L. casei*: 0<br>*L. paracasei*: 100<br>*L. rhamnosus*: 27<br>*L. zeae*: 0	 99–100<br>98.62–100	 100<br>100
LGG_00444	SpaC, VWA domain-containing protein, pilus specific protein, ancillary protein involved in mucus-adhesion	*L. casei*: 0<br>*L. paracasei*: 100<br>*L. rhamnosus*: 27	 93.73 – 99.4<br>97.47–100	 100<br>100

		L. zeae: 0		
LGG_00500	MetQ, D-methionine-binding lipoprotein, surface antigen	*L. casei*: 100<br>*L. paracasei*: 100<br>*L. rhamnosus*: 100<br>*L. zeae*: 100	73.51–74.13<br>76.13–75.13<br>97.44–100<br>75.41–75.51	89<br>57–86<br>100<br>85–86
LGG_00501	Bsh, choloylglycine hydrolase, bile salt hydrolase	*L. casei*: 100<br>*L. paracasei*: 100<br>*L. rhamnosus*: 100<br>*L. zeae*: 100	79.65–79.94<br>76.38–76.97<br>97.24–100<br>79.65–80.73	100<br>99<br>100<br>100
LGG_00503	Myosin-cross-reactive antigen family protein with structural features in common with the beta chain of the class II antigens	*L. casei*: 100<br>*L. paracasei*: 100<br>*L. rhamnosus*: 100<br>*L. zeae*: 100	84.37–85.01<br>81.87–82.36<br>96.93–100<br>84.95–85.18	100<br>100<br>100<br>99
LGG_00544	TauE, protein involved in the export of sulfoacetate during taurine metabolism	*L. casei*: 100<br>*L. paracasei*: 0<br>*L. rhamnosus*: 100<br>*L. zeae*: 100	74.37–74.87<br><br>96.14 – 100<br>74.90–75.56	100<br><br>99–100<br>100
LGG_00576	WxL domain containing protein	*L. casei*: 93<br>*L. paracasei*: 96<br>*L. rhamnosus*: 100<br>*L. zeae*: 50	75.83–76.58<br>75.77–76.5<br>94.43–100<br>76.42	93<br>95<br>100<br>93
LGG_00578	Cell surface protein with LPxTG motif for cell wall anchoring by sortases, peptidase	*L. casei*: 0<br>*L. paracasei*: 75<br>*L. rhamnosus*: 100<br>*L. zeae*: 0	 72.95–73.77<br>90.48–100 	 33<br>100
LGG_00579	Lectin-like protein, WxL domain	*L. casei*: 0<br>*L. paracasei*: 0<br>*L. rhamnosus*: 100<br>*L. zeae*: 0	 <br>80.82–100 	 <br>73–100
LGG_00583	WxL domain containing protein	*L. casei*: 0<br>*L. paracasei*: 96<br>*L. rhamnosus*: 78	 69.00–73.54<br>97.63–100	 97–98<br>100

		L. zeae: 0		
LGG_00584	Cell surface protein with LPxTG motif for cell wall anchoring by sortases	*L. casei*: 0<br>*L. paracasei*: 0<br>*L. rhamnosus*: 81<br>*L. zeae*: 0	 <br>95.47–100 	 <br>100
LGG_00585	WxL domain-containing protein, Llp1 lectin-like protein	*L. casei*: 0<br>*L. paracasei*: 92<br>*L. rhamnosus*: 81<br>*L. zeae*: 0	 69.82–71.01<br>97.68–100 	 88<br>100
LGG_00587	WxL domain-containing protein, lectin-like protein 1	*L. casei*: 0<br>*L. paracasei*: 94<br>*L. rhamnosus*: 81<br>*L. zeae*: 0	 67.18–69.43<br>97.40–100 	 94<br>100
LGG_00645	Glycosyltransferase, group 2, a paralog is plasmid encoded in some *L. paracasei* strains	*L. casei*: 100<br>*L. paracasei*: 100<br>*L. rhamnosus*: 100<br>*L. zeae*: 100	72.63–74.55<br>73.76–74.81<br>96.57–100<br>72.52–72.48	90<br>90<br>100<br>90
LGG_00695	GtrB, glycosyltransferase, group 2	*L. casei*: 100<br>*L. paracasei*: 94<br>*L. rhamnosus*: 100<br>*L. zeae*: 100	76.28–77.20<br>73.29–73.69<br>98.47–100<br>76.51–76.71	100<br>99<br>100<br>100
LGG_00696	YkcB, 4-amino-4-deoxy-L-arabinose transferase related glycosyltransferase of PMT family protein	*L. casei*: 100<br>*L. paracasei*: 94<br>*L. rhamnosus*: 100<br>*L. zeae*: 100	75.03–81.50<br>74.07–78.52<br>96.47–100<br>75.20–81.14	88–100<br>84<br>100<br>91–100
LGG_00697	GtcA family protein, involved in lipoteichoic acid (LTA) glycosylation	*L. casei*: 100<br>*L. paracasei*: 94<br>*L. rhamnosus*: 100<br>*L. zeae*: 100	77.21–86.49<br>76.47–77.45<br>99.75–100<br>85.75–86.49	99–100<br>100<br>100<br>99
LGG_00825	RfaG, glycosyltransferase group 1	*L. casei*: 100<br>*L. paracasei*: 100<br>*L. rhamnosus*: 100	81.97–83.29<br>81.01–80.61<br>99.67–100	100<br>100<br>100

		L. zeae: 100	83.03–83.53	100
LGG_00826	CpoA, glycosyltransferase group 1	*L. casei*: 100	80.47–80.86	100
		L. paracasei: 100	78.52–78.91	100
		L. rhamnosus: 100	98.25–100	100
		L. zeae: 100	80.27–80.66	100
LGG_00827	Flippase-like domain containing protein	*L. casei*: 100	80.72–81.02	99
		L. paracasei: 100	78.60–79.10	100
		L. rhamnosus: 100	98.62–100	100
		L. zeae: 100	80.52–81.22	99
LGG_00830	LTA synthase family protein	*L. casei*: 100	81.05–81.34	98–99
		L. paracasei: 100	79.07–79.60	99
		L. rhamnosus: 100	98.62–100	100
		L. zeae: 100	81.09–81.29	99
LGG_00851	Polysaccharide biosynthesis protein, transport membrane protein	*L. casei*: 100	81.82–81.95	100
		L. paracasei: 100	79.93–83.35	100
		L. rhamnosus: 100	98.18–100	100
		L. zeae: 100	81.46	100
LGG_00914	LiaX, daptomycin-sensing surface protein, virulence factor	*L. casei*: 100	80.12–80.46	99
		L. paracasei: 100	78.48–78.81	100
		L. rhamnosus: 100	98.05–100	100
		L. zeae: 100	80.59–80.86	99
LGG_00998	ArbX, lipopolysaccharide biosynthesis glycosyltransferase	*L. casei*: 100	75.95–76.55	100
		L. paracasei: 100	72.50–72.86	99
		L. rhamnosus: 100	97.86–100	100
		L. zeae: 100	76.44	99
LGG_00999	ArbY, lipopolysaccharide biosynthesis glycosyltransferase	*L. casei*: 100	77.46–78.31	98
		L. paracasei: 100	74.74–75.5	98
		L. rhamnosus: 100	96.97–100	100
		L. zeae: 100	77.25–77.35	98
LGG_01002	SpdI family membrane protein, immunity protein that protects toxin-producing cells from being killed	*L. casei*: 100	79.80–80.81	100
		L. paracasei: 100	75.00–76.52	100
		L. rhamnosus: 100	100	98.99–100

		L. zeae: 100	80.05–81.06	100
LGG_01057	Glycosyltransferase group 2	*L. casei*: 100	77.66–78.33	99
		L. paracasei: 100	73.18–74.36	95
		L. rhamnosus: 100	95.32–100	100
		L. zeae: 100	77.76–77.85	99
LGG_01066	YhfO family protein, possible glycosyltransferase of the PTM group	*L. casei*: 100	71.45–71.92	93–97
		L. paracasei: 100	71.94–72.54	95–97
		L. rhamnosus: 100	96.90–100	100
		L. zeae: 100	71.39–71.60	93
LGG_01069	GtrB, glycosyltransferase group 2	*L. casei*: 100	75.73–77.25	98–99
		L. paracasei: 100	76.82–77.58	99
		L. rhamnosus: 100	96.77–100	100
		L. zeae: 100	75.16–75.76	98
LGG_01147	Glycosyltransferase group 1, also on plasmid for 5 *L. paracasei* strains	*L. casei*: 100	81.01–81.79	100
		L. paracasei: 100	78.12–78.72	100
		L. rhamnosus: 100	97.77–100	100
		L. zeae: 100	80.84–81.19	100
LGG_01366	Lipopolysaccharide assembly LapA domain containing protein	*L. casei*: 100	76.76–77.37	100
		L. paracasei: 100	75.59–76.25	91
		L. rhamnosus: 100	97.55–100	99–100
		L. zeae: 100	77.98	100
LGG_01450	FbpA, fibronectin binding protein A	*L. casei*: 100	80.36–80.58	100
		L. paracasei: 100	78.86–78.33	99
		L. rhamnosus: 100	97.77–100	100
		L. zeae: 100	80.40–80.81	100
LGG_01538	Glycosyltransferase group 2, phage related	*L. casei*: 40	99.47	99
		L. paracasei: 1	99.73	99
		L. rhamnosus: 27	99.60–100	99–100
		L. zeae: 0		
LGG_01586	YohH, glycosyltransferase, group 1	*L. casei*: 100	65.09–66.84	36–55
		L. paracasei: 32.7	77.20–77.40	99
		L. rhamnosus: 100	75.39–100	99–100

		L. zeae: 100	64.81–64.98	49
LGG_01587	YohJ, glycosyltransferase, group 1	*L. casei*: 0<br>*L. paracasei*: 32.7<br>*L. rhamnosus*: 100<br>*L. zeae*: 0	 75.72–77.71<br>70.55–100	 94–99<br>89–100
LGG_01589	CAP superfamily, cysteine-rich secretory proteins, antigen 5 and pathogenesis-related, surface-docked cell-binding protein SpcA	*L. casei*: 0<br>*L. paracasei*: 28<br>*L. rhamnosus*: 84<br>*L. zeae*: 0	 69.05–69.12<br>95.75–100	 56<br>88–100
LGG_01590	Ig-like protein, fibrinogen-binding MSCRAMM adhesin Fss2	*L. casei*: 0<br>*L. paracasei*: 28<br>*L. rhamnosus*: 93<br>*L. zeae*: 0	 67.50<br>97.08–100	 83<br>100
LGG_01591	KxYKxGKxW signal peptide domain-containing protein, FliK family flagellar hook-length control protein, Ig-like domain containing protein	*L. casei*: 100<br>*L. paracasei*: 34<br>*L. rhamnosus*: 93<br>*L. zeae*: 100	65.50–68.34<br>70.65–70.98<br>97.43–100<br>66.73–67.2	22–28<br>63–78<br>100<br>21–25
LGG_01592	SpcB, Ser-Ala-175 repeat glycoprotein adhesin SpcB, docking protein that forms a complex on the surface with the SpcA protein	*L. casei*: 0<br>*L. paracasei*: 34<br>*L. rhamnosus*: 93<br>*L. zeae*: 0	 66.84–67.47<br>96.14–100	 82–97<br>99–100
LGG_01765	WxL domain containing protein	*L. casei*: 0<br>*L. paracasei*: 100<br>*L. rhamnosus*: 100<br>*L. zeae*: 0	 95.56–100<br>64.59–65.25	 100<br>88
LGG_01827	AI-2E family transporter, involvement in biofilm formation	*L. casei*: 100<br>*L. paracasei*: 100<br>*L. rhamnosus*: 100<br>*L. zeae*: 100	79.32–80.34<br>77.48–78.02<br>98.56–100<br>79.49–79.58	100<br>93<br>100<br>100
LGG_01865	MabA conserved extracellular matrix binding protein	*L. casei*: 100<br>*L. paracasei*: 0<br>*L. rhamnosus*: 100	65.78–66.15<br><br>90.02–100	93–95<br><br>100

		L. zeae: 100	66.27–66.40	93–95
LGG_01877	FeoB, membrane protein essential for Fe(II) uptake in prokaryotes	*L. casei*: 60	78.24–79.41	99
		L. paracasei: 100	71.76–72.35	99
		L. rhamnosus: 100	98.25–100	100
		L. zeae: 100	78.24–78.36	99-100
LGG_01883	MucBP mucus binding domain-containing protein	*L. casei*: 100	70.70–71.34	96
		L. paracasei: 100	66.81–67.86	96
		L. rhamnosus: 100	97.75–100	100
		L. zeae: 100	70.91	96
LGG_01990*	Polysaccharide deacetylase	*L. casei*: 100	75.81–76.32	69
		L. paracasei: 100	72.16–72.78	69 -72
		L. rhamnosus: 100	71.85–100	68–100
		L. zeae: 100	74.39–75	69–71
LGG_02016	Surface antigen NLP/P60, C40 family peptidase	*L. casei*: 100	76.89–77.92	100
		L. paracasei: 100	70.66–71.57	100
		L. rhamnosus: 100	97.50–100	100
		L. zeae: 100	77.31–77.72	100
LGG_02036*	Wzb, Capsular polysaccharide biosynthesis protein	*L. casei*: 100	81.26–82.18	99
		L. paracasei: 100	80.65–82.07	99–100
		L. rhamnosus: 100	90.21–100	100
		L. zeae: 100	81.78–82.44	99
LGG_02144	GtcA, cell wall teichoic acid glycosylation protein, putative flippase	*L. casei*: 100	81.71–81.95	99
		L. paracasei: 100	77.53–79.26	98
		L. rhamnosus: 100	97.81–100	100
		L. zeae: 100	80 – 80.98	99
LGG_02282	LPXTG cell wall anchor domain-containing protein, KxYKxGKxW signal peptide domain-containing protein, peptidase, cell surface protein, fibrinogen binding	*L. casei*: 100	65.80–66.77	91
		L. paracasei: 0		
		L. rhamnosus: 100	95.54–100	100
		L. zeae: 100	66.05–68.08	73–89
LGG_02337	MucBP mucin binding domain-containing protein, InlJ internalin	*L. casei*: 100	63.62–65.23	52–57
		L. paracasei: 30	67.86 – 69.9	31–34
		L. rhamnosus: 100	96.65–100	99–100

		L. zeae: 100	64.15–64.55	48–60
LGG_02369	SrtC2, sortase C	*L. casei*: 0<br>*L. paracasei*: 100<br>*L. rhamnosus*: 100<br>*L. zeae*: 0	 70.49–71.00<br>95.36–100	 95<br>99–100
LGG_02370	SpaH/EbpB family LPXTG-anchored major pilin, SpaD	*L. casei*: 0<br>*L. paracasei*: 100<br>*L. rhamnosus*: 100<br>*L. zeae*: 0	 69.88–70.39<br>96.27–100	 95–99<br>100
LGG_02371	SpaE, pilus specific protein, minor backbone protein	*L. casei*: 0<br>*L. paracasei*: 100<br>*L. rhamnosus*: 100<br>*L. zeae*: 0	 80–85<br>97.71–100	 72.86–73.88<br>100
LGG_02372	LPXTG cell wall anchor domain-containing protein, SpaF, pilus specific protein, ancillary protein involved in adhesion	*L. casei*: 0<br>*L. paracasei*: 100<br>*L. rhamnosus*: 100<br>*L. zeae*: 0	 66.08–70.17<br>96.48–100	 67–82<br>100
LGG_02423	PsaA, putative adhesion lipoprotein, zinc ABC transporter, surface adhesin	*L. casei*: 100<br>*L. paracasei*: 100<br>*L. rhamnosus*: 100<br>*L. zeae*: 100	80.64–80.75<br>78.35 -78.81<br>98.04–100<br>80.75–80.97	99<br>98<br>100<br>99
LGG_02426	PsaA, putative adhesion lipoprotein, zinc ABC transporter, surface adhesin	*L. casei*: 100<br>*L. paracasei*: 100<br>*L. rhamnosus*: 100<br>*L. zeae*: 100	75.69–76.35<br>75.85–76.53<br>98.67–100<br>75.91–75.94	100<br>87–98<br>100<br>100
LGG_02520	Polysaccharide transport membrane protein	*L. casei*: 100<br>*L. paracasei*: 100<br>*L. rhamnosus*: 100<br>*L. zeae*: 100	76.88–77.41<br>75.52–76.03<br>97.84–100<br>75.98	100<br>100<br>100<br>100
LGG_02652	α-L-fucosidase	*L. casei*: 14<br>*L. paracasei*: 58<br>*L. rhamnosus*: 44	77.22<br>78.12–78.30<br>98.08–100	99<br>99<br>100

		L. zeae: 50	75.05	99
LGG_02734	Cell envelope-associated proteinase, lactocepin PrtR	*L. casei*: 100	77.85–78.91	36–86
		L. paracasei: 0		
		L. rhamnosus: 100	96.28–100	100
		L. zeae: 100	76.51–78.46	88–95
LGG_02869	Glycosyltransferase group 1	*L. casei*: 100	81.99–82.35	98
		L. paracasei: 100	77.33–78.45	99
		L. rhamnosus: 100	96.93–100	100
		L. zeae: 50	81.62–82.44	98
LGG_02923	Adhesion exoprotein	*L. casei*: 0		
		L. paracasei: 100	67.64–70.58	44–78
		L. rhamnosus: 94	76.69–100	99–100
		L. zeae: 0		

* initial ORF in EPS production gene clusters variable in gene presence/absence, succession and function as shown in Figure 1.
